# Clinical associations and related factors of metabolic syndrome in systemic sclerosis: results from an observational multicenter study of GIRRCS (Gruppo Italiano di Ricerca in Reumatologia Clinica e Sperimentale)

**DOI:** 10.1007/s00296-026-06100-9

**Published:** 2026-06-05

**Authors:** Vasiliki Liakouli, Giulio Forte, Piero Ruscitti, Marco Minerba, Antonio Orlando, Luca Navarini, Francesca Bellisai, Francesco Caso, Giuliana Guggino, Lidia La Barbera, Chiara Rizzo, Ada Corrado, Paola Triggianese, Alberto Lo Gullo, Giuseppe Mandraffino, Luca Cantarini, Bruno Frediani, Paola Cipriani, Francesco Paolo Cantatore, Maria Sole Chimenti, Elvira Favoino, Federico Perosa, Annamaria Iagnocco, Roberto Giacomelli, Francesco Ciccia

**Affiliations:** 1 Rheumatology Unit, Department of Precision Medicine, University of Campania “Luigi Vanvitelli”, Naples, Italy; 2 Department of Biotechnological and Applied Clinical Sciences, University of L’Aquila, L’Aquila, Italy; 3 Rheumatology and Clinical Immunology, Department of Medicine, School of Medicine, University of Rome Campus Biomedico, Rome, Italy; 4 Leeds Institute of Rheumatic and Musculoskeletal Medicine, University of Leeds, Leeds, UK; 5 Clinical and Research Section of Rheumatology and Clinical Immunology, Fondazione Policlinico Campus Biomedico, Rome, Italy; 6 Rheumatology Unit, Azienda Ospedaliero Universitaria Senese, Siena, Italy; 7 Rheumatology Unit, Department of Clinical Medicine and Surgery, University of Naples Federico II, Naples, Italy; 8 Department of Health Promotion, Mother and Child Care, Internal Medicine and Medical Specialties, Rheumatology section, University of Palermo, Sicily, Italy; 9 Rheumatology Clinic, Department of Medical and Surgical Sciences, University of Foggia, Foggia, Italy; 10 Department of Biomedicine and Prevention, University of Rome Tor Vergata, Rome, Italy; 11 UOSD Rheumatology, Papardo Hospital, Messina, Italy; 12 Internal Medicine Unit, University Hospital G. Martino, University of Messina, Messina, Italy; 13 Rheumatology Unit, Department of Medicine, Surgery and Neurosciences, University of Siena, Siena, Italy; 14 Rheumatology, Allergology and Clinical Immunology, Department of “Medicina dei Sistemi”, University of Rome Tor Vergata, Rome, Italy; 15 Rheumatic and Systemic Autoimmune Diseases Unit, Department of Interdisciplinary Medicine (DIM), University of Bari Medical School, Bari, Italy; 16 Academic Rheumatology Centre, Department of Clinical and Biological Science, Hospital Mauriziano, University of Turin, Turin, Italy

**Keywords:** Scleroderma, systemic, Metabolic syndrome, Hypertension, pulmonary, Capillaroscopy, nailfold, Lung disease, interstitial

## Abstract

Metabolic syndrome (MetS) has been increasingly recognised as a relevant comorbidity in systemic autoimmune diseases; however, data on its prevalence and clinical associations in systemic sclerosis (SSc) remain limited and heterogeneous. This study aimed to evaluate the prevalence of MetS in a large Italian SSc cohort and to explore its clinical, functional, and vascular associations. A cross-sectional multicenter study enrolled 613 SSc patients from 11 tertiary Rheumatology centres across Italy, fulfilling ACR/EULAR 2013 criteria. MetS was defined according to the International Harmonised 2009 Joint Interim Statement criteria. Clinical, laboratory, functional, and imaging data were collected. Comparisons were performed between MetS+ve and MetS-ve patients. Logistic regression analysis was performed to identify factors associated with MetS. Among 570 patients with complete data, MetS was identified in 8.4% (48/570). MetS+ve patients were older and more frequently had active disease according to the EScSG index (45.2% vs. 17.7%, *p* = 0.0001). They more commonly exhibited mild restrictive lung impairment, with a higher proportion showing FVC 70–79% (21.6% vs. 7%, *p* = 0.008), as well as moderately reduced DLCO. Cardiovascular involvement was more prevalent in MetS+ve patients, particularly left ventricular diastolic dysfunction (54.3% vs. 25.8%, *p* = 0.0001) and clinically diagnosed PAH (27.3% vs. 12.6%, *p* = 0.007). Moreover, advanced microvascular damage, reflected by a higher frequency of late nailfold videocapillaroscopy (NVC) pattern (38.5% vs. 23.1%, *p* = 0.031), was more common among MetS+ve patients. In multivariable analysis adjusted \for age and sex, active disease (OR 4.79, 95% CI 2.09–10.98; *p* < 0.0001) and mild lung restriction (FVC 70–79%) (OR 4.54, 95% CI 1.62–12.69; *p* = 0.004) remained independently associated with MetS. Systemic arterial hypertension was the most frequent component of MetS (77.1%). In this large Italian SSc cohort, MetS, although relatively infrequent, identified a distinct clinical phenotype characterised by increased disease activity, mild lung functional impairment, and more advanced microvascular damage. These findings support the clinical relevance of cardiometabolic comorbidity in SSc and highlight the importance of comprehensive metabolic assessment in routine care. Prospective studies are needed to clarify temporal relationships and underlying mechanisms.

**Supplementary Information:**

The online version contains supplementary material available at 10.1007/s00296-026-06100-9.

## Introduction

Metabolic syndrome (MetS), characterised by insulin resistance, central obesity, dyslipidemia, and hypertension, is increasingly recognised as a relevant comorbidity in systemic autoimmune diseases, including systemic sclerosis (SSc) [1]. In patients with SSc, the presence of MetS is associated with increased cardiovascular risk, higher mortality, and greater disease severity [2, 3].

In the general Italian population, the prevalence of MetS varies widely according to age, ranging from approximately 3–5% in individuals aged 20–29 years to 25–30% in those aged ≥ 65 years [4]. Similarly, a large survey of adult outpatients followed by general practitioners across Italy reported a MetS prevalence of 33.0%, with abdominal obesity and elevated blood pressure representing the most frequent components [5]. Although data specifically addressing Italian SSc cohorts are limited, available studies suggest that MetS affects between 8.4% and 19% of patients with SSc [1–6].

From a pathophysiological perspective, MetS has been suggested to be associated with SSc through partially overlapping mechanisms. Low-grade inflammation, oxidative stress, endothelial dysfunction, and alterations in metabolic homeostasis have been described in SSc and may be relevant in this context. In line with this background, metabolomic studies have reported changes in amino acid, lipid, and energy metabolism in SSc, in association with immune activation, microvascular abnormalities, fibrosis, and gut microbiome dysregulation [7].

Despite growing interest in cardiometabolic comorbidities in SSc, previous investigations, including those conducted within the GIRRCS network, have mainly focused on individual cardiovascular risk factors or specific organ involvement. A comprehensive evaluation of MetS as an integrated clinical entity, and its association with disease activity, cardiopulmonary manifestations, and microvascular damage assessed by nailfold videocapillaroscopy (NVC), remains limited. Therefore, the present multicenter study aimed to address this gap by evaluating the prevalence of MetS in a large Italian SSc cohort and by systematically investigating its clinical, functional, and vascular correlates.

## Methods

### Study design, patients, and assessment of MetS

The study population included 613 SSc patients from 11 tertiary rheumatology units across Italy. All patients fulfilled the American College of Rheumatology/European League Against Rheumatism (ACR/EULAR) 2013 classification criteria [8] were consecutively enrolled from January 1, 2021, to February 15, 2023. Inclusion criteria were age ≥ 18 years, fulfilment of the 2013 ACR/EULAR classification criteria for SSc, and availability of comprehensive clinical, serological, disease activity, and laboratory data necessary for MetS classification according to the International Harmonised 2009 Joint Interim Statement criteria (JIS) at the time of assessment. No additional exclusion criteria were applied, except for overlap connective tissue diseases, active malignancy, pregnancy, or absence of essential data required for the analysis. The Ethics Committee of the coordinator of the study approved the protocol (0029176/I, approval date: November 30, 2020, CET Campania 2) following the Good Clinical Practice Guidelines and the Declaration of Helsinki (October 2024 revision). Written informed consent was obtained from all the patients.

The sample size was determined by consecutive enrolment of all eligible patients attending the participating centres during the predefined study period. Given the observational and cross-sectional design of the study, no formal a priori sample size calculation was performed.

This study was conducted and reported in accordance with the Strengthening the Reporting of Observational Studies in Epidemiology (STROBE) guidelines for cross-sectional studies.All patients underwent a detailed medical history, physical examination, and comprehensive laboratory and radiological assessment related to SSc and MetS. MetS was defined according to the harmonised 2009 Joint Interim Statement criteria (JIS), which require the presence of at least three of the following five components: elevated waist circumference (ethnicity-specific cut-offs), elevated triglycerides (≥ 150 mg/dL) or specific treatment for hypertriglyceridemia, reduced HDL cholesterol (< 40 mg/dL in men or < 50 mg/dL in women) or specific treatment, elevated blood pressure (systolic ≥ 130 mmHg and/or diastolic ≥ 85 mmHg) or antihypertensive treatment, and elevated fasting plasma glucose (≥ 100 mg/dL) or previously diagnosed type 2 diabetes [9]. Pharmacological treatment for hypertension, dyslipidemia, or hyperglycemia was considered as fulfilling the corresponding diagnostic criterion, in accordance with the harmonized 2009 JISdefinition, The BMI was used to categorise patients as underweight (BMI < 18.5 kg/m2), normal weight (BMI 18.5–25 kg/m2), overweight (BMI 25–30 kg/m2), or obese (BMI > 30 kg/m2). Clinical and disease features collected included disease duration from first non-Raynaud’s symptom, SSc subset, anti-nuclear antibodies (ANA) and SSc-specific autoantibodies, digital ulcers, calcinosis, tendon friction rubs, renal crisis, pulmonary function tests, interstitial lung disease (ILD) assessed by high-resolution computed tomography (HRCT), ECG, echocardiography, modified Rodnan skin score (mRSS). PAH was confirmed by right heart catheterisation (RHC) when available. In the absence of RHC, PAH was defined based on clinical evaluation and non-invasive investigations. Specifically, an estimated systolic pulmonary arterial pressure (sPAP) > 40 mmHg on echocardiography was considered suggestive of pulmonary hypertension, when associated with compatible clinical features (symptoms of dyspnoea, reduced DLCO, or other signs suggestive of pulmonary vascular involvement) and after exclusion of significant left heart disease or advanced interstitial lung diseases. Due to the cross-sectional design, RHC was not systematically performed in all patients.Disease activity was assessed using the European Scleroderma Study Group (EScSG) activity index. Patients were classified as having active disease when the EScSG score was ≥ 3, according to the original definition [9]. Lung restriction was categorised by TLC (≥ 80%, 70–79%, 50–69%, < 50%), adapted from the ATS/ERS guidelines for restrictive lung patterns [10], and moderate groups were combined into a single group to ensure an adequate sample size for statistical analysis. Current therapy was also recorded. NVC was performed according to standardised procedures using a videocapillaroscope with 200× magnification, and capillaroscopic patterns were classified as early, active, or late according to the Cutolo classification [11].

All the researchers followed a unified assessment protocol grounded in standardised clinical definitions and validated evaluation tools. Data collection was centrally reviewed for accuracy and completeness. Any inconsistencies or missing information were promptly addressed through direct coordination with the contributing centres. All assessments were performed by experienced clinicians.

### Statistical analysis

Data were collected and analysed using SPSS software, version 20 (SPSS Inc., Chicago, IL, USA). The distribution of continuous variables was assessed using the Shapiro-Wilk test. Continuous variables are reported as mean ± standard deviation (SD) or median with interquartile range (IQR), as appropriate, while categorical variables are presented as absolute frequencies and percentages. Comparisons between MetS+ve and MetS-ve patients were performed using the Mann-Whitney U test for continuous variables and the chi-square test for categorical variables.

A total of 613 patients were enrolled in the study. Of the 613 enrolled patients, complete data required for the classification of MetS were available for 570 patients, who constituted the analytical sample for descriptive and univariate analyses. The 43 patients with incomplete MetS data were excluded from these analyses. Univariate logistic regression analyses were performed to explore associations between metabolic syndrome and SSc-related clinical features using available complete cases for each variable.

Given the limited number of MetS+ve cases, a parsimonious multivariable logistic regression was subsequently performed using a complete-case approach, including only patients with complete data for all covariates entered into the model (*n* = 373). No imputation procedures were applied.

Variables directly related to the definition or clinical management of MetS (including antihypertensive agents, ACE inhibitors, lipid-lowering therapies, and antidiabetic medications) were not included in the multivariable models to avoid overadjustment, as pharmacological treatment is part of the diagnostic criteria. Odds ratios (ORs) with 95% confidence intervals (CIs) were reported. A two-tailed p-value < 0.05 was considered statistically significant. The selection of patients for descriptive, univariate, and multivariable analyses is detailed in Supplementary **Figure S1.**

## Results

### Baseline characteristics of the evaluated SSc patients

Among patients with complete data for MetS classification, 48 patients fulfilled the criteria for MetS, corresponding to a prevalence of 8.4% **(**Table 1**).** MetS+ve SSc patients were significantly older (64.81 ± 10.33 vs. 59.55 ± 14.13 years; *p* = 0.006) and had higher frequencies of positive ANA antibodies (93.5% vs. 81.7%, *p* = 0.044). Although disease duration and subset distribution were similar, MetS+ve SSc patients showed greater disease activity, with 45.2% classified as active by the EScSG index compared to 17.7% in MetS-ve (*p* = 0.0001). Lung function impairment was more frequently observed among MetS+ve patients, as reflected by a lower proportion maintaining normal FVC values (≥ 80%) compared with MetS-ve patients (70.3% vs. 86.3%, *p* = 0.008). Notably, this difference was mainly driven by a higher proportion of MetS+ve patients exhibiting mildly reduced lung volumes, with 21.6% having an FVC between 70 and 79%. Cardiovascular involvement was more frequently observed among MetS+ve patients, with a higher prevalence of left ventricular diastolic dysfunction compared with MetS-ve patients (54.3% vs. 25.8%, *p* = 0.0001). In descriptive analyses, PAH, defined on the basis of clinical evaluation and available diagnostic investigations, was also more prevalent among MetS+ve patients (27.3% vs. 12.6%, *p* = 0.007) (Table 1).

**Table 1:** Main demographic and disease-related characteristics of the study population

Variable	SSc patients (*n* = 613)	MetS-ve SSc patients (*n* = 522)	MetS+ve SSc patients (*n* = 48)	*P* value (MetS-ve vs. MetS+ve)
Gender (Female)	560 (91.4)	479 (91.9)	42 (8.1)	0.287
Age mean ± SD (years)	**59.53 ± 13.93**	**59.55 ± 14.13**	**64.81 ± 10.33**	**0.006**
Disease duration from non-RP mean ± SD (years)	12.51 ± 11.67	11.61 ± 11.99	11.14 ± 10.25	0.542
BMI				
Normal	**435 (74.4)**	**424 (81.7)**	**5 (10.6)**	**0.0001**
Overweight	**106 (18.1)**	**76 (14.6)**	**24 (51.1)**	**0.0001**
Obese	**44 (7.5)**	**19 (3.7)**	**18 (38.3)**	**0.0001**
dcSSc	171 (28)	15 (26.3)	18 (37.5)	0.095
ANA (positive)	**503 (83.4)**	**421 (81.7)**	**43 (93.5)**	**0.044**
ACA (positive)	277 (47.1)	243 (48.2)	19 (41.3)	0.369
ATA (positive)	190 (31)	161 (30.9)	16 (33.3)	0.728
ARA (positive)	16 (2.6)	13 (2.5)	2 (4.2)	0.488
ACA/ATA/ARA (negative)	106 (18)	88 (17.5)	9 (19.6)	0.720
Puffy fingers	292 (47.9)	241 (46.3)	28 (58.3)	0.109
Ischemic digital ulcers	236 (38.7)	199 (38.2)	20 (41.7)	0.636
Pitting scars	179 (30.3)	157 (30.7)	16 (34)	0.855
Telangiectasias	288 (49.3)	257 (50.9)	17 (36.2)	0.054
Calcinosis	76 (12.9)	69 (13.5)	3(6.4)	0.162
Tendon friction rubs	68 (11.2)	56 (10.8)	6 (12.5)	0.713
EScSG (active)	**86 (19.5)**	**72 (17.7)**	**14 (45.2)**	**0.0001**
NVC pattern				
Early	131 (24.3)	113 (23.9)	7 (17.9)	0.396
Active	206 (38.2)	188 (39.8)	12 (30.8)	0.265
Late	**129 (23.9)**	**109 (23.1)**	**15 (38.5)**	**0.031**
SRC	9 (1.5)	9 (1.7)	0 (0)	0.359
FVC%				
≥ 80%	**429 (84.6)**	**385 (86.3)**	**26 (70.3)**	**0.008**
70–79%	**41 (8.1)**	**31 (7)**	**8 (21.6)**	**0.006**
50–69	36 (7.1)	30 (6.7)	2 (5.4)	1.000
< 50%	1 (0.2)	0 (0)	1 (2.7)	0.077
O2 therapy	0 (0)	0 (0)	0 (0)	-
FEV1/FVC < 80%	112 (25.7)	89 (23.1)	11 (34.4)	0.152
DLCO%				
≥ 80%	**209 (44.6)**	**195 (47.3)**	**6 (17.6)**	**0.001**
70–79%	81 (17.3)	70 (17)	9 (26.5)	0.164
50–69%	**128 (27.3)**	**105 (25.5)**	**15 (44.1)**	**0.019**
< 50%	51 (10.9)	42 (10.2)	4 (11.8)	0.768
TLC%				
≥ 80%	287 (76.1)	253 (77.1)	17 (63)	0.097
70–79%	45 (11.9)	40 (12.2)	4 (14.8)	0.759
50–69%	34 (9)	27 (8.2)	5 (18.5)	0.082
< 50%	11 (2.9)	8 (2.4)	1 (3.7)	0.513
Conduction abnormalities	76 (15.2)	62 (14.5)	11 (25)	0.066
Arrhythmias	35 (7)	28 (6.5)	4 (9.1)	0.522
RV hypertrophy	6 (1.2)	5 (1.2)	1 (2.3)	0.466
EF < 55%	14 (2.5)	12 (12.5)	1 (2.2)	1.000
Diastolic dysfunction	**164 (29)**	**128 (25.8)**	**25 (54.3)**	**0.0001**
Pericardial effusion	81 (13.9)	73 (14.4)	3 (6.5)	0.137
PAH (RHC)†	**75 (14.7)**	**57 (12.6)**	**12 (27.3)**	**0.007**
mRSS > 14	87 (17.5)	71 (16.6)	10 (25)	0.176
ILD	272 (46.9)	226 (45.1)	26 (56.5)	0.137
ESR>30mmhg	**134 (25.8)**	**101 (22.5)**	**23 (51.1)**	**0.0001**

Demographic and clinical characteristics of SSc patients with and without MetS. Data refers to patients with complete information for MetS classification according to the 2009 harmonised JIS criteria (*n* = 570)

*BMI* body mass index, *ACA* anticentromere antibodies, *ANA* antinuclear antibodies, *ARA* Anti-RNA polymerase III, *ATA* anti-topoisomerase I antibodies, *CRP* C reactive protein, *DLCO* Diffusing capacity for carbon monoxide, *ECG* elettrocardiogram, * EF* ejection fraction, *ESR* erythrocyte sedimentation rate, *EScSG* European Scleroderma Study Group activity index, *FVC* Forced vital capacity, *ILD* interstitial lung disease, *mRSS* modified skin score, *NVC* nailfold videocapillaroscopy, *PAH* pulmonary arterial hypertension,* PAPs* pulmonary arterial pressure, *PPI* proton pump inhibitors,*RP* Raynaud’s phenomenon,*TLC* total lung capacity

†PAH was defined only in patients with PAH confirmed by RHC. RHC was performed in a subset of patients according to clinical indications

RHC was performed only in a subset of patients according to clinical indications. Consequently, the number of RHC-confirmed PAH cases was lower than the overall number of clinically diagnosed PAH cases. Within the complete-case population used for multivariable regression analyses, no MetS+ve patients had PAH confirmed by RHC; therefore, this variable could not be included in the multivariable model.

NVC patterns were similar except for a higher frequency of the late pattern (38.5% vs. 23.1%, *p* = 0.031) in MetS+ve patients, suggesting advanced microvascular damage. MetS+ve patients more frequently exhibited elevated inflammatory markers, as reflected by higher ESR values (> 30 mm/h) and BMI distribution (normal: 1.6% vs. 81.7%; overweight: 51.1% vs. 14.6%; obese: 38.3% vs. 3.7%; all *p* = 0.0001). Immunosuppressive and vasoactive treatment was similar between groups, except that MetS+ve patients more frequently received antihypertensive including ACE inhibitors, beta-blockers, diuretics, and statins (Table S1). Lastly, among patients fulfilling the criteria for MetS, systemic arterial hypertension was the predominant component (77.1%), with more than half of patients also exhibiting reduced HDL cholesterol (52.5%) or elevated triglycerides (51.2%), while type 2 diabetes was present in 43.8% of cases.

### Factors associated with MetS of the evaluated SSc patients

Univariate analysis showed an association of MetS with several SSc-related features (Table 2). A significant association was observed with age (OR: 1.033, 95% CI: 1.009–1.059, *p* = 0.008), mildly reduced FVC (70–79%) (OR: 3.693, 95% CI: 1.557–8.759, *p* = 0.003), moderately reduced DLCO (50–69%) (OR: 2.308, 95% CI 1.132–4.706, *p* = 0.021), left ventricular diastolic dysfunction (OR: 3.432, 95% CI: 1.857–6.342, *p* = 0.0001), PAH (RHC) (OR: 2.612, 95% CI: 1.272–5.362; *p* = 0.009), active disease (EScSG) (OR: 3.832, 95% CI: 1.807–8.126; *p* = 0.0001), NVC late pattern (OR: 2.081, 95% CI: 1.055–4.107; *p* = 0.035). Conversely, preserved lung function (FVC ≥ 80%) (OR: 0.374, 95% CI 0.176–0.797, *p* = 0.011), and higher DLCO (> 80%) (OR: 0.238, 95% CI 0.097–0.588, *p* = 0.002) were associated with a lower likelihood of MetS in SSc patients. In a parsimonious multivariable logistic regression model adjusted for age and sex, EScSG-defined active disease (EScSG ≥ 3) and mild restrictive lung impairment (FVC 70–79%) remained independently associated with the presence of MetS (Table 3). Specifically, active disease was associated with an almost 5-fold higher odds of MetS (OR: 4.79, 95% CI: 2.09–10.98; *p* < 0.0001), while patients with FVC 70–79% had a similarly increased likelihood of MetS (OR: 4.54, 95% CI: 1.62–12.69; *p* = 0.004). Age and sex were not significantly associated with MetS in the adjusted model.

**Table 2:** MetS univariate analysis in SSc patients

Variable	OR	*P*	CI 95%
Gender	0.628	0.317	0.253–1.562
Age	**1.033**	**0.008**	**1.009–1.059**
RP onset	1.001	0.927	0.978–1.025
Non-RP onset	1.006	0.661	0.979–1.034
dcSSc	1.682	0.098	0.908–3.114
ANA+ve	3.200	0.056	0.972–10.536
ACA+ve	0.756	0.370	0.410–1.394
ATA+ve	1.118	0.728	0.597–2.095
RNA Pol III+ve	1.702	0.492	0.373–7.776
ACA/ATA/Pol III (negative)	1.116	0.589	0.750–1.659
Puffy fingers	1.627	0.112	0.893–2.961
Ischemic digital ulcers	1.156	0.637	0.634–2.107
Pitting scars	0.981	0.872	0.774–1.242
Telangiectasias	0.547	0.056	0.294–1.017
Calcinosis	0.436	0.174	0.132–1.442
Tendon friction rubs	1.184	0.713	0.482–2.909
SRC	-	**-**	-
FVC%			
≥ 80%	**0.374**	**0.011**	**0.176–0.797**
70–79%	**3.693**	**0.003**	**1.557–8.759**
50–69%	0.792	0.757	0.182–3.454
< 50%	-	-	-
DLCO%			
> 80%	**0.238**	**0.002**	**0.097–0.588**
70–79%	1.759	0.169	0.787–3.931
50–69%	**2.308**	**0.021**	**1.132–4.706**
< 50%	1.175	0.772	0.395–3.497
TLC%			
> 80%	0.504	0.102	0.221–1.147
70–79%	1.252	0.692	0.412–3.808
50–69%	2.534	0.082	0.889–7.225
< 50%	1.538	0.690	0.185–12.778
FEV1/FVC%	1.742	0.156	0.809–3.751
Conduction abnormalities	1.968	0.071	0.945–4.098
Arrhythmias	1.429	0.524	0.477–4.278
RV hypertrophy	1.967	0.541	0.225–17.229
Diastolic dysfunction	**3.432**	**0.0001**	**1.857–6.342**
Pericardial effusion (PE)	0.415	0.149	0.125–1.372
PAH (RHC)†	**2.612**	**0.009**	**1.272–5.362**
ILD	1.311	0.434	0.666–2.580
EScSG (active)	**3.832**	**0.0001**	**1.807–8.126**
NVC pattern			
Early	0.695	0.398	0.299–1.617
Active	0.671	0.268	0.332–1.358
Late pattern	**2.081**	**0.035**	**1.055–4.107**
ESR>30mmHg	**3.592**	**0.0001**	**1.922–6.711**

Univariate logistic regression analysis was performed using available complete cases for each variable

ACA: anticentromere antibodies; ANA: antinuclear antibodies; ARA: Anti-RNA polymerase III; ATA: anti-topoisomerase I antibodies; CRP: C reactive protein; CVD: cardiovascular disease; dcSSc: diffuse cutaneous systemic sclerosis; DLCO: Diffusing capacity for carbon monoxide; EF: ejection fraction; ESR: erythrocyte sedimentation rate; EScSG: European Scleroderma Study Group activity index; FVC: Forced vital capacity; FEV1/FVC: Forced Expiratory Volume in the first second to forced vital capacity; IFG: impaired fasting glucose; ILD: interstitial lung disease; mRSS: modified Rodnan Skin Score;; NVC: nailfold videocapillaroscopy; PAH: pulmonary arterial hypertension; PAPs: estimated pulmonary arterial pressure by echocardiography; RHC: right heart catheterization; RP: Raynaud’s phenomenon; SRC: scleroderma renal crisis; TLC: total lung capacity

†PAH confirmed by RHC was not included in the multivariable model due to the absence of RHC-confirmed PAH cases among MetS+ve patients within the complete-case population used for regression analyses

**Table 3:** MetS multivariate analysis in SSc patients

Variable	OR	*P*	95% CI
Gender	1.017	0.981	0.258–4.015
Age	1.019	0.284	0.985–1.054
EScSG	**4.788**	**0.0001**	**2.088–10.979**
FVC 70–79%	**4.537**	**0.004**	**1.622–12.690**

Multivariable logistic regression analysis was performed on patients with complete data for all covariates included in the model (*n* = 373)

* EScSG* European Scleroderma Study Group activity index, *FVC* Forced vital capacity, *FVC* Forced Vital Capacity

## Discussion

In our large multicenter cohort of 570 patients with SSc, we observed a prevalence of MetS of 8.4%, as defined by the2009 harmonised JIS criteria. MetS was significantly associated with higher disease activity (EScSG ≥ 3), mild restrictive ventilatory impairment (FVC 70–79%), and a greater frequency of late scleroderma pattern at NVC, indicative of advanced microvascular damage. Despite its relatively low prevalence, MetS identified a distinct subgroup of patients characterised by higher disease activity and mild impairment of pulmonary function, suggesting a potential clinical relevance in SSc. This prevalence aligns with prior data from Italian cohorts, where reported MetS rates ranged between 8.4% and 19% [1, 6].

In contrast, substantially higher rates have been observed in non-European studies, including 36.4% in a Mexican SSc population [12]. These discrepancies may reflect geographic and demographic differences, including age, BMI distribution, and background cardiovascular risk, as well as the diagnostic criteria employed.

Importantly, beyond prevalence estimates, our study provides novel insights into the clinical correlates of MetS in SSc. In both univariate and multivariable models, MetS was independently associated with active disease and mildly impaired lung function. This reinforces the hypothesis of a pathophysiological link between systemic inflammation and metabolic dysregulation in autoimmune diseases [13]. Similar findings have been reported in previous studies, in which SSc patients with metabolic syndrome exhibited higher disease severity scores, suggesting a possible amplifying effect of metabolic dysfunction on overall disease burden [1]. In contrast, other investigations have reported more limited clinical differences between MetS-positive and MetS-negative SSc patients, with associations confined mainly to specific manifestations such as an increased prevalence of cardiac arrhythmias [6], underscoring the need for further well-designed studies to clarify these relationships.

A particularly novel contribution of our study is the observed association between MetS and late NVC patterns. While NVC is well-established as a non-invasive marker of SSc-related microangiopathy and disease severity, its association with systemic metabolic status has not been previously described in depth. Our finding that patients with MetS more frequently exhibit late NVC patterns may suggest a potential synergistic interplay between metabolic dysfunction and advanced microvascular damage. Both conditions share common pathogenetic mechanisms, including endothelial dysfunction, oxidative stress, chronic low-grade inflammation, and impaired nitric oxide bioavailability, which may converge at the microvascular level and contribute to capillary loss and structural vascular abnormalities characteristic of the late scleroderma pattern. From a clinical perspective, this association may indicate that metabolic comorbidity identifies a subgroup of SSc patients with more pronounced vascular involvement and potentially an increased cardiopulmonary risk profile. Furthermore, pulmonary involvement, a major contributor to morbidity in SSc, was characterised by a higher frequency of lung function impairment among MetS+ve patients, with a greater proportion exhibiting mild restrictive changes. These findings indicate an association between MetS and reduced lung volumes; however, they should be interpreted with caution. Mildly reduced FVC may reflect, at least in part, the mechanical effects of central obesity and body habitus rather than more severe intrinsic pulmonary involvement. However, large population-based data support an association between MetS, particularly abdominal obesity, and impaired lung function [14–17].

This study has several limitations that should be acknowledged. First, the cross-sectional design precludes causal inference regarding the relationships between MetS and SSc-related features, and the temporal direction of these associations cannot be established. Second, the study population was recruited from tertiary referral centres of Italy, which may introduce a selection bias toward patients with more severe or complex disease. Therefore, the findings may not be fully generalizable to non-Italian populations or to patients managed in primary or secondary care settings. In addition, the limited number of patients fulfilling MetS criteria restricted the statistical power for subgroup analyses and may have prevented the detection of additional associations with less common clinical manifestations. Furthermore, PAH was defined based on clinical evaluation and available investigations, while RHC was performed only when clinically indicated. As a result, RHC confirmation was not available for all patients, and no MetS+ve patients had RHC-confirmed PAH, potentially leading to misclassification and limiting the interpretation of this association.

Despite these limitations, the study has several notable strengths. It represents one of the largest cohorts specifically evaluating metabolic syndrome in SSc, allowing for a robust assessment of its prevalence and clinical correlates. Moreover, the comprehensive and standardised clinical phenotyping, including disease activity, pulmonary and cardiovascular involvement, and NVC, enabled an integrated analysis of metabolic, immunological, functional, and microvascular features. The use of validated classification criteria and standardised assessment tools further strengthens the internal consistency and reliability of the findings.

Overall, MetS identifies a distinct clinical phenotype in a subset of patients with SSc, characterised by higher disease activity and greater organ involvement. Although causality cannot be inferred, these findings support an integrated clinical approach addressing both autoimmune and metabolic aspects of the disease. Longitudinal and mechanistic studies are needed to clarify the temporal relationships and underlying pathways.

## Supplementary Information

Below is the link to the electronic supplementary material.

Supplementary Material 1

Supplementary Material 2

Supplementary Material 3
