# Supplementary material for: Clinical associations and related factors of metabolic syndrome in systemic sclerosis: results from an observational multicenter study of GIRRCS (Gruppo Italiano di Ricerca in Reumatologia Clinica e Sperimentale)
Source: Rheumatol Int. 2026 Jun 5;46(6):132. doi: 10.1007/s00296-026-06100-9 (PMC13241409; doi:10.1007/s00296-026-06100-9)
Supplement: Supplementary file 1 — Supplementary Material 1 [file 296_2026_6100_MOESM1_ESM.docx]

# STROBE Checklist – Cross‑Sectional Study

Manuscript: Clinical Associations and Related Factors of Metabolic Syndrome in Systemic Sclerosis

| Item | Recommendation | Manuscript location |
| --- | --- | --- |
| 1 | Study design indicated in title/abstract | Title; Abstract Methods |
| 2 | Scientific background explained | Introduction paragraphs 1–3 |
| 3 | Objectives clearly stated | Introduction final paragraph |
| 4 | Study design presented early | Methods – Study design |
| 5 | Setting and dates described | Methods – Study design and patients |
| 6 | Eligibility criteria described | Methods – Study design and patients |
| 7 | Variables clearly defined | Methods – Assessment of MetS; Clinical variables |
| 8 | Data sources/measurement | Methods – Clinical and laboratory assessment |
| 9 | Bias addressed | Discussion – Limitations |
| 10 | Study size explained | Methods – Sample size paragraph |
| 11 | Handling of quantitative variables | Methods – Statistical analysis |
| 12 | Statistical methods | Methods – Statistical analysis |
| 13 | Participants numbers reported | Results; Figure S1 flowchart |
| 14 | Descriptive data | Results; Table 1 |
| 15 | Outcome data | Results section |
| 16 | Main results (adjusted/unadjusted) | Results; Tables 2–3 |
| 17 | Other analyses | Results – components of MetS |
| 18 | Key results summarised | Discussion paragraph 1 |
| 19 | Limitations discussed | Discussion – Limitations |
| 20 | Interpretation | Discussion final paragraphs |
| 21 | Generalisability | Discussion – Limitations |
| 22 | Funding sources | Declarations – Funding |
