# Supplementary material for: Clinical associations and related factors of metabolic syndrome in systemic sclerosis: results from an observational multicenter study of GIRRCS (Gruppo Italiano di Ricerca in Reumatologia Clinica e Sperimentale)
Source: Rheumatol Int. 2026 Jun 5;46(6):132. doi: 10.1007/s00296-026-06100-9 (PMC13241409; doi:10.1007/s00296-026-06100-9)
Supplement: Supplementary file 2 — Supplementary Material 2 [file 296_2026_6100_MOESM2_ESM.docx]

**Figure S1.** Flow chart of patient selection and analytical samples.

**Figure S1.** Of 613 enrolled patients with SSc, 570 had complete data for MetS classification according to the 2009 harmonised JIS criteria and were included in prevalence estimates and descriptive analyses. Multivariable logistic regression analysis was conducted using a complete-case approach and therefore included only patients with complete data for all covariates entered in the final model (age, sex, EScSG activity index, and FVC category).
