# Supplementary material for: Clinical associations and related factors of metabolic syndrome in systemic sclerosis: results from an observational multicenter study of GIRRCS (Gruppo Italiano di Ricerca in Reumatologia Clinica e Sperimentale)
Source: Rheumatol Int. 2026 Jun 5;46(6):132. doi: 10.1007/s00296-026-06100-9 (PMC13241409; doi:10.1007/s00296-026-06100-9)
Supplement: Supplementary file 3 — Supplementary Material 3 [file 296_2026_6100_MOESM3_ESM.docx]

**Table S1.** Treatment characteristics of the study population stratified by metabolic syndrome status

| **Variable** | **SSc patients**  **(n=613)** | **MetS-ve SSc patients**  **(n=522)** | **MetS+ve SSc patients**  **(n=48)** | **P value**  **(MetS-ve vs MetS+ve)** |
| --- | --- | --- | --- | --- |
| MMF | 90 (15.6) | 83 (15.6) | 7 (15.9) | 0.953 |
| AZA | 37 (6.4) | 34 (6.4) | 3 (6.8) | 0.912 |
| MTX | 54 (9.2) | 48 (9) | 5 (11.4) | 0.603 |
| HCQ | 84 (14.2) | 74 (13.9) | 6 (13.6) | 0.964 |
| GCs | 179 (30.3) | 160 (30) | 14 (31.89) | 0.803 |
| Colchicine | 9 (1.5) | 7 (1.3) | 2 (4.5) | 0.097 |
| RTX | 15 (2.5) | 14 (2.6) | 0 (0) | 0.276 |
| TCZ | 9 (1.55) | 8 (1.5) | 1 (2.3) | 0.694 |
| Antifibrotics | 3 (0.5) | 2 (0.4) | 0 (0) | 0.684 |
| CCB | 345 (58.5) | 316 (59.3) | 23 (52.3) | 0.364 |
| Iloprost | 224 (38) | 206 (38.6) | 12 (27.3) | 0.135 |
| Tadalafil | 4 (0.7) | 15 (2.8) | 3 (6.8) | 0.142 |
| Riociguat | 3 (0.5) | 3 (0.5) | 0 (0) | 1.000 |
| Bosentan | 139 (23.6) | 128 (24.6) | 11 (8.2) | 0.842 |
| Macitentan | 12 (2) | 12 (2) | 0 (0) | 1.000 |
| Ambrisentan | 2 (0.3) | 2 (0.3) | 0 (0) | 1.000 |
| Antiplatelet | 324 (54.9) | 298 (55.9) | 23 (52.3) | 0.641 |
| Anticoagulant | 19 (3.2) | 16 (3) | 3 (6.8) | 0.174 |
| ACEi | 77 (13.1) | 65 (12.2) | 12 (27.3) | **0.005** |
| ARB | 46 (7.8) | 40 (7.5) | 5 (11.1) | 0.359 |
| **β-blockers** | **51 (8.6)** | **35 (6.6)** | **16 (36.4)** | **0.001** |
| **Diuretics** | **67 (11.4)** | **53 (9.9)** | **13 (29.5)** | **0.001** |
| **Statins** | **103 (17.5)** | **89 (16.7)** | **13 (29.5)** | **0.032** |

**Table S1**: Summary of pharmacological treatments in SSc patients stratified by metabolic syndrome status. Treatments are expressed as numbers and percentages.

**Abbreviations**: MMF: mycophenolate mofetil; AZA: azathioprine; MTX: methotrexate; HCQ: hydroxychloroquine; GC: glucocorticoids; RTX: rituximab; TCZ: tocilizumab; CCB: calcium channel blockers; ACEi: angiotensin converting enzyme inhibitors; ARB: angiotensin 2 receptor blocker.
